# A high level estimation of the net economic benefits to small-scale livestock producers arising from animal health product distribution initiatives

**DOI:** 10.3389/fvets.2023.1171989

**Published:** 2023-06-06

**Authors:** Paul R. Bessell, Gareth Salmon, Christian Schnier, Katharine Tjasink, Lamyaa Al-Riyami, Andrew Peters

**Affiliations:** ^1^Independent Consultant, Edinburgh, United Kingdom; ^2^SEBI-L Supporting Evidence Based Interventions in Livestock, University of Edinburgh, Edinburgh, United Kingdom; ^3^Global Alliance for Livestock Veterinary Medicines (GALVmed), Edinburgh, United Kingdom

**Keywords:** animal health, veterinary pharmaceutical, intervention, model, livestock disease

## Abstract

**Introduction:**

A fundamental challenge for charities that facilitate distribution of animal health products to small-scale livestock producers (SSPs) in low and middle income countries (LMICs) is identifying the products and market mechanisms that provide the greatest positive impact for SSPs and estimating their associated impact. This paper describes a pragmatic approach to modeling the impact of market-led product distribution initiatives based on estimating the net economic benefit of administration of animal health products.

**Methods:**

The model estimates the economic impact of diseases at the individual animal level for poultry, small ruminants, and cattle. The economic impact of mortality and growth inhibition associated with disease are then estimated in conjunction with the losses averted or recovered by preventing or treating the disease. Economic benefit is estimated in 2014–2017 values and also adjusted to 2023 values. The flexible model structure allows for addition of new geographies, new products, and increased granularity of modeled production systems.

**Results:**

Applied to the Global Alliance for Livestock Veterinary Medicines (GALVmed) product distribution initiatives conducted in Africa and South Asia (SA) between 2014 and 2017, the model estimates an adjusted total net economic benefit of 139.9 million USD from sales of vaccines and poultry anthelminthics in these initiatives. Within SSA, the greatest net economic benefit was realized from East Coast fever and Newcastle disease vaccines, while in SA, peste des petits ruminants and Newcastle disease vaccines had the greatest net economic benefits. This translated to an adjusted $37.97 of net economic benefit on average per SSP customer, many of whom were small poultry producers.

**Discussion:**

While the model currently estimates impacts from mortality and growth inhibition in livestock, there is the potential to extend it to cover impacts of further initiatives, including interventions targeted at diseases that impact production of milk, eggs, and reproduction.

## 1. Introduction

The significant contribution of livestock farming to supporting livelihoods of people in low and middle income countries (LMICs) is widely recognized (1). Estimates suggest that around 1 billion of the world’s poorest people depend on livestock for income generation, as savings assets, for non-marketable products such as draft power and manure (2) to support crop production and as a direct source of nutrition (3, 4). Livestock farming presents an opportunity to enter agricultural output markets and to participate in the informal sector (5). Beyond the farm-level, livelihoods can be improved through the creation of employment and other benefits across livestock value chains (6, 7). Livestock has the potential to fuel local non-farm job creation, for example through agro-industry, and contribute toward an inclusive and diverse rural non-farm economy, with ripple effects into the local economy (8, 9). There is also a growing body of evidence demonstrating that livestock offer, or have the potential to offer, a unique resource for women; improving gender equality through ownership of assets, decision making and control of income (10, 11). As such, the improvement of livestock productivity within small scale producer settings can make a positive contribution to many of the Sustainable Development Goals (SDGs) (12, 13). Notably: SDG1, no poverty; SDG2, zero hunger; SDG3 good health and wellbeing; and SDG5, gender equality. However, this production is frustrated by several factors with poor animal health and losses due to disease being key constraints alongside sub-optimal animal genetics and inadequate nutrition (14). Specifically, the estimated annual cost of livestock mortality alone due to disease in Africa exceeds 9 billion US dollars (USD) which equates to 6% of the total value of the livestock sector in Africa (15).

For many livestock diseases, veterinary drugs (referred to in this paper as “animal health products” or “products”) are available for prevention or treatment. The Food and Agriculture Organization of the United Nations (FAO) defines veterinary drugs as “drugs, insecticides, vaccines and biological products, used or presented as suitable for use, to prevent, treat, control or eradicate animal pests or diseases, or to be given to animals to establish a veterinary diagnosis, or to restore, correct or modify organic functions” (16). Animal health products have the potential to create direct economic benefits for SSPs with consequential effects for human welfare improvement. Econometric modeling of the effects of East Coast fever (ECF) vaccination found that vaccination contributes to net income and this additional income is directed into food purchases and child education (17). A randomized control trial on the effects of poultry vaccination found that the use of vaccination and concomitant reduction in chicken mortality was causally related to improved height-for-age as a direct result of children’s increased consumption of protein and foods rich in macronutrients (18).

While suitable animal health products are commercially available for many diseases, a recent analysis of animal health product use in Africa found that the average utilization of animal health products per unit of livestock in smallholder poultry is between 12 and 50 percent of the average world use level (19). While overuse of animal health products and particularly antibiotics is a serious problem (20) there remains a challenge getting critical product classes to particular sectors. As a result, disease, caused by infectious agents, has a devastating effect on livestock health. The ability of SSPs, in particular, to access suitable animal health products is limited by multiple factors including a lack of knowledge, lack of extension services, high product and treatment prices, poor product efficacy, inappropriate product pack sizes, and inconvenient storage requirements for some products (21). In addition, particularly in Africa and South Asia (SA), multiple structural factors affect the veterinary drug supply chain and limit SSP access to these products (19).

Efforts to better control livestock disease in SSP systems can come in the form of public services with the cost of interventions covered by governments and non-governmental organizations (NGOs). However, some consider that this can inhibit private investment and sustainable market development (22). Commercially anchored product and market development initiatives are a potentially sustainable and scalable alternative. Some (23, 24), such as the Global Alliance for Livestock Veterinary Medicine (GALVmed), take this alternative approach with the objective of using market forces to improve the uptake of both newly developed and existing animal health products. The work of GALVmed enables improved distribution and access to existing products as well as research and development for new animal health products to meet the needs of the SSP market (12). Through partnerships with the animal health industry, GALVmed initiatives make appropriate products available to SSPs at market prices through accessible retailers as well as an expanding network of animal health professionals. Such initiatives show evidence of positive impacts in terms of improving productivity (16–18).

Although some studies have explored the effect of controlling livestock disease on livestock output and asset values (25–27), there is a general paucity of information on the economic benefits of preventive and curative animal health products for SSPs. This gap in knowledge is a problem for investments in the livestock sector and more specifically in animal health. In response, the Supporting Evidence based Interventions-Livestock (SEBI-L) initiative has partnered with GALVmed to develop a model, specifically for practical use by GALVmed and its partners, with the aim of prioritizing product development decisions and directing market development efforts. Such a broad strategic assessment is novel and has hitherto been unavailable to GALVmed and other organizations working on behalf of the SSP sector.

To address important questions concerning impact, a modeling framework was developed considering the animal health products that are being sold to SSPs and administered to animals, enabled through GALVmed initiatives. As SSPs are using these products to prevent or ameliorate loss due to disease, we model the key ways in which those losses are experienced by farmers and estimate the proportion of those losses that are averted by using the product. This provides an estimate of net economic benefit (NEB) to the individual SSP. When combined with the total number of SSPs served by an initiative (which can reach millions) this gives the total NEB. By comparing total NEB generated through various approaches, GALVmed can readily compare these approaches at a high level to determine which strategies are working best in the field.

## 2. Materials and methods

In this paper, we use a model to estimate the impact of products sold during the GALVmed People and Livelihoods 2 (PL2) initiatives between 2014 and 2017 in Africa and SA. The PL2 initiative supported manufacturers in product production and distribution of poultry anthelminthics (PA) and vaccines against Newcastle disease (ND), fowl pox (FP), sheep and goat pox (SGP), peste des petits ruminants (PPR) and ECF. These are applied to sheep, goats, and backyard chickens in SA and Africa, and cattle in Africa alone as ECF is the only cattle vaccine in the PL2 initiative and its use was restricted to Africa. Distribution to SSPs was facilitated through networks of trained and supported vaccinators, comprising either lay vaccinators with basic training in administering poultry vaccines and anthelminthics (18) or animal health professionals in the case of ECF (28). Therefore products were not sold through retail outlets, but via these intermediaries.

**Figure 1:**
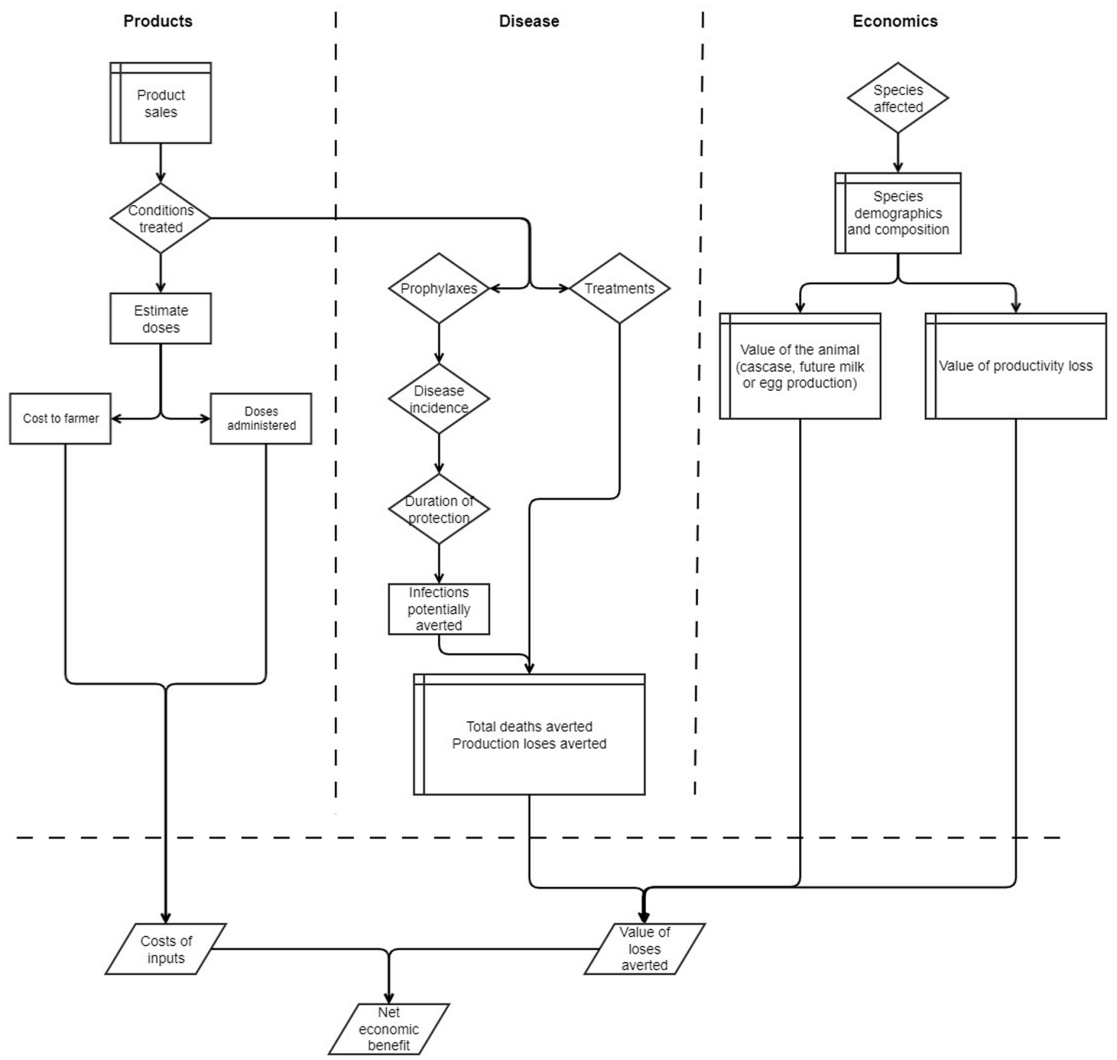
Swim-lane diagram of the impact model illustrating the three components for product sales, the disease epidemiology, and the associated economics. Table shapes represent input data, diamonds parameters, rectangles processes, and parallelograms output results.

A schematic diagram of the model framework is shown in Figure 1. The model is conceptualized in terms of three components: products, disease epidemiology, and economics. The products component includes sales of animal health products and number of animals that are forecast to be treated with different pack sizes. The disease epidemiology component comprises the conditions that are treated using the products, number of infections, mortality rates and impact on growth rate. The economics component comprises various measures of losses arising from reduced level of production and direct losses from livestock mortality that are averted by using the product. The economics component is finally merged with the other components to measure the NEB from use of animal health products. While here we use the model to estimate benefits arising from use of vaccines and anthelminthics associated with GALVmed’s PL2 initiative, we also demonstrate how the model can be used to estimate impact from using other treatments.

Starting with the animal health product sales (Figure 1), we consider the conditions or diseases that are treated or prevented by using the product. We then estimate the number of doses in a packet, the cost to the farmer, and the number of doses that are administered to animals. During the PL2 initiative, the products were sold to SSPs by vets, animal health workers, and village vaccinators through outreach programs and so the cost of administration to the animal is implicit in the modeled costs. However, costs to the farmer related to gathering animals for vaccination are not included. If the model were to be applied to a product purchased and administered by the SSP, then the cost of purchase and administration should be factored in as well as any losses due to pack sizes not matching the SSP’s requirements.

The conditions treated or prevented continues into the disease epidemiology component, where the products are either classified as prophylaxes or treatments. For treatments, we assume that the animal being treated has the condition and therefore requires intervention. For prophylaxes, we assume that there is some probability of the animal becoming infected, and that this is either a function of disease incidence and duration of protection from the vaccination to give the numbers of infections potentially averted, or a function of the prevalence of infections for the number treated for helminth infections. For both treatments and prophylaxes, the impact of mortalities and reduced levels of production from these infections are estimated.

For the economics component, we consider the species that are affected by the disease, their demographics and typical composition to estimate the averted mortalities and production losses. The economics are then combined with disease production losses and products sold to estimate an economic benefit.

### 2.1. Level of analysis

The model operates at the level of specific products administered to animals for the prevention of disease. In these analyses, we consider only vaccines that are used to prevent or reduce the risk/impact of specific diseases and poultry anthelminthics which are typically administered *en masse* akin to a poultry vaccine. Based on these, the model estimates the economic benefit realized by the SSP who has used the animal health product on their livestock.

### 2.2. Products covered

In the PL2 initiative, the products used include vaccines against FP, ND, PPR, SGP (including a combination SGP-PPR vaccine) and an ECF vaccine in addition to PA (Table 1). Sales are based on records maintained by the distributors and reporting to GALVmed.

**Table 1:** Products sales and costs to farmers disaggregated by Africa and SA regions.

Product	Species	Doses sold and reported to GALVmed (2014–2017)		Pricing and delivery	
		Africa	SA	Cost per dose (USD)	Typical pack (or vial) size
FP vaccine	Poultry	399,770	3,084,167	0.03	200
ND vaccine	Poultry	55,501,674	113,612,156	0.03	200
PA	Poultry	2,072,936	8,191,251	0.036	125
PPR vaccine	SR	2,013,837	2,209,333	0.16	100
SGP vaccine	SR	–	3,636,200	0.21	100
SGP and PPR vaccine	SR	116,584	–	0.26	100
ECF vaccine	Cattle	1,271,872	–	8	40

FP, Fowl pox; ND, Newcastle disease; PA, poultry anthelminthics; PPR, Peste des petits ruminants; SGP, sheep and goat pox; ECF, East Coast fever; SR, Small ruminants (sheep and goats); SA, South Asia.

Data on the costs of products are informed by reported field estimates and represent the administered cost when products are administered by a third party. The values are reported by the distribution partners (Table 1). Note that costs of ECF vaccines vary depending on location and size of herd and animals (29).

### 2.3. Epidemiological model

The underlying epidemiological model is a loss envelope (30). In this model there is a certain maximum level of production that can be achieved without a disease and then a minimum level of production that is achieved with untreated disease. In the model, we estimate the resulting level of production once the disease is treated or prevented and then the difference between this value and the minimum represents the gross economic benefit of using the product.

The model is at the level of the product, so we consider whether the treated animal has the disease, or whether it would have acquired an infection during the period of protection by the prophylaxis. We consider the following parameters in evaluating this:

- The period between outbreaks of a disease, which for endemic production diseases was defined as the time to reinfection in years (r_interepi_).
- The annual incidence of disease during outbreaks (r_incidence_).
- The duration of protection of the vaccine (years of protection, or lifelong protection) (y_protection_).
- The animal’s life expectancy in years (r_animal_), assuming that the animal is treated at the mid-point of its life.

The sources of the parameters are described in later sections. We use these parameters to calculate the probability that an animal would be infected during the period of time when the animal is protected by the animal health product (*p_animal_*):


$$p_{animal}=1-{\left(1-r_{incidence}.y_{protection}\right)}^{\frac{1}{r_{interepi}}}$$

if protection is not lifelong


$$p_{animal}=\frac{1-{\left(1-r_{incidence}\right)}^{\frac{r_{animal}}{2}}}{r_{interepi}}$$

if protection is lifelong, with the assumption that the animal is vaccinated at the midpoint of life.

We then consider the efficacy of the product which describes whether the product will successfully prevent disease or treat the infection.

### 2.4. Measuring “disease caused” production impact

The production impairment due to a disease is measured in terms of the following components.

Mortality of infected animals, measured as a case fatality rate.

Impact on animal growth and development (meat production modeled as the number of weeks that the development of the animal to adult weight is delayed).

Due to the nature of the diseases covered under this product portfolio, we do not currently consider the acute, short-term impacts on milk production or fertility and abortion resulting from morbidity caused by these diseases. However, in estimating the value of animal mortality we include the future lost milk and egg production. These could be bought into future iterations of the model depending on diseases and products covered in future GALVmed initiatives.

### 2.5. Parameter estimation

The parameters used in this model are described below and were initially derived from a review by J. Mariner (Pers. Comm), which are verified and adjusted by separate parameter scoping. The values assigned in Table 2 focus on incidence, mortality rates and impairments to productivity. Productivity impairments are often more difficult to estimate. The product efficacies presented in Table 2 describe the extent to which vaccines prevent infections and adverse effects. Generally PPR and SGP vaccines have high protective efficacy (31, 32). For ECF there is an association of ECF infection with reduced milk yield. In this model, while the economics of the value of the animal consider future lifetime milk production in the value of the animal, we do not here model an acute loss of milk. With regards to poultry diseases the same applies to egg production due to the often low levels of egg off-take in backyard sectors, but there could be an impact on production not considered here (18, 33–35).

**Table 2:** Summary of parameter values used in the model with sensitivity range of 50–200% of the baseline value.

Disease/infection	Annual incidence	Case fatxality proportion	Duration of protection	Production Impairment (weeks of impairment per infection)	Interepidemic period (years between outbreaks)	Modeled product efficacy^1^
	Proportion (Range)	Proportion (Range)	Years	Weeks (Range)	Years	Percentage
PA	0.736 (0.373–1.0)	0.002 (0.001–0.004)	0.25	4 (2–8)	0.25	80%
ND	0.42 (0.21–0.84)	0.34 (0.17–0.68)	0.33	2 (1–4)	1	95%
FP	0.2 (0.1–0.4)	0.15 (0.075–0.3)	Lifelong	4 (2–8)	1	95%
PPR	0.2 (0.1–0.4)	0.132 (0.066–0.264)	Lifelong	2 (1–4)	1	98%
SGP	0.5 (0.25–1.0)	0.08 (0.04–0.16)	Lifelong	2 (1–4)	2.5	98%
ECF	0.25 (0.13–0.5)	0.21 (0.11–0.42)	Lifelong	4 (2–8)	1	98%

PA, Poultry anthelminthics; ND, Newcastle disease; FP, Fowl pox; PPR, Peste des petits ruminants; SGP, Sheep and goat pox; ECF, East Coast fever.

^1^Product efficacy, the proportion of doses that infer effective protection against disease. Here we model the impact of a single product dose and consider the protection given during that period.

#### 2.5.1. Helminth infestation–poultry

A meta-analysis of helminth infections in poultry has estimated the prevalence in backyard systems at 73.6% (4.39–100%) (36) with regular reinfection. As the range of species of helminths that are found in backyard indigenous systems is high (36–40) we allow that anthelminthics are effective against only 80% of infections depending on treatment and infection (41). There is a marked beneficial impact on chicken growth from administration of anthelminthics (42), where among young stock (40–70 days) the estimated impact of treatment was a further 90 g over the 8 weeks following treatment. As the animals are growing at 68 g per week during this period, and assuming that not all birds will be infected in this younger stock (we allow 1/3 of those treated would have had infection cleared), the 90 g additional growth converts to 4 weeks of growth among all infected stock. We also allow for a small rate of mortality arising from infection.

#### 2.5.2. Newcastle disease

While the morbidity and mortality rates from ND can be very high, this is hugely variable owing to the range of virus types that circulate. Furthermore, *postmortem* investigations of ND cases in backyard settings are few, so causes of death are often unconfirmed (43, 44). Indeed, the relatively large numbers of reported outbreaks following vaccination underline that ND may be over-diagnosed in backyard settings (18). Hence here we are using a relatively lower, but still high overall, annual mortality rate of 14.3% among the population. This is consistent with seroprevalence estimates of 40% [albeit with potentially large amounts of low-pathogenic strains (45)], with ND accounting for 33% of sick chickens (46). We allow a small impairment for production and a slightly reduced efficacy because the vaccines may not be effective against locally circulating strains (46, 47). In backyard settings, revaccination is recommended every 3 to 4 months to ensure protection and outbreaks of ND are typically annual and seasonal (47, 48), hence we assume annual outbreaks and 4 months of vaccine protection.

#### 2.5.3. Fowl pox

Data on the impact of FP in backyard production are very limited. Outbreaks are irregular, can be associated with high mortality, and have production impairments (49). Biswas et al. reported 9.8% of overall poultry mortality was due to FP (50). By assuming an overall mortality rate of 33% backyard poultry, we here adopted an overall annual mortality rate (incidence x case fatality rate) of 3% (50) based on a case fatality rate of 15% and annual incidence of 20%. Production losses of 4 weeks reflect the typical disease course and vaccines are typically efficacious, but there are some escapes (49, 51).

#### 2.5.4. Peste des petits ruminants

The parameters for PPR are based on a meta-analysis conducted by Mariner et al. (52). The estimates that are reported in the meta-analysis have subsequently been adjusted because the parameters report the overall burden of disease whereas the vaccine sales, and therefore this model, explicitly target those herds that are regularly infected. Consequently, we rule out those that have infrequent outbreaks that are included in Mariner et al. (52). While the duration of protection of the vaccine is estimated to be in the region of 4 years relative to the lifespan of a small ruminant, we assumed this to be lifelong (53) and the coverage of viral lineages is good giving a high vaccine efficacy (53). Outbreaks are not typically seen annually owing to the immune status of animals and the lower susceptibility of non-naïve populations (32). Due to the acute nature of infection, the period of impairment for infected animals is typically 2 weeks (32).

#### 2.5.5. Sheep and goat pox

While infection rates are generally high for SGP, the mortality rates are typically below 10% in endemic areas (31, 54, 55), although higher in sheep than in goats and can be considerably greater than 10% (56). There are occasional outbreaks of pox viruses (57), so we estimate 50% outbreak incidence with 2.5 years between outbreaks. We also assume an impairment to productivity (55, 56).

#### 2.5.6. East coast fever

There is huge variation in ECF, particularly with breeds and animal ages. Bronsvoort et al. (58) found 16.1% mortality in animals under 1 year with 40% attributed to *T. parva*. In a smaller study of calves, the annual incidence rate was 48% among non-immunized animals with 21% case fatality (59). Here we use the 48% incidence extrapolated to give 25% annual incidence averaged over the first 4 years of an animal’s life with 21% case fatality rate. Kivaria et al. found similar mortality rates to ECF in grazed systems (60). Immunity to infection following inoculation is typically lifelong and as the vaccine covers the majority of strains it is effective at preventing infection and disease, with 98% efficacy (59, 61). Owing to the high morbidity associated with ECF infection, and long recovery period, the period of production impairment is set to 4 weeks. While there is an acute impact of ECF on milk production (62), we do not model this here due to the small off-take by beef producers who are the main users of ECF vaccines (29, 63).

#### 2.5.7. Peste des petits ruminants and SGP combination vaccine

The parameters for the PPR and SGP vaccine must be adjusted to prevent double counting. Therefore, the parameters for incidence and mortality have been generated using the compound probability.

The parameters that have been identified (Table 2) are indicative, and often conservative, but in reality, vary depending on a range of factors, such as disease strain, production system setting, breed, stress levels of the animal and past exposure to the pathogen. In Table 2, we include the ranges used in sensitivity analyses to illustrate the impact of varying the parameter between 50 and 200% of its baseline value.

### 2.6. Economic model components

#### 2.6.1. Livestock systems

Included in the framework are beef cattle, sheep, goats, and backyard chickens (Table 3). These are the key groups that were targeted through the PL2 initiative of GALVmed whereby SSPs were targeted through local vaccinators (18). In the current model, these are presented as discrete species groups but, in reality, these species groups can be much more nuanced. We have not modeled dairy systems at this stage because, while the ECF vaccines were also sold to dairy producers, a majority were sold to beef producers due to issues of practicality and greater prevalence (29, 63). However, the sales data do not allow the separation of sales from beef producers from those sales to dairy SSPs.

**Table 3:** Breakdown of the livestock species groups included in this model.

Livestock species sector	Description
Cattle	Indigenous and improved cattle breeds reared primarily for beef. While there is often a small amount of milk taken from such animals in Africa, we focus on the primary commercial purpose of the animal, so any potential milk off-take is not considered. The Africa production systems targeted through the PL2 initiative included many pastoralist cattle herders adopting the product. For such pastoralists, who largely own indigenous cattle breeds (such as Zebu, Boran, Fulani, Ankole), we are assuming 8 year life expectancy for the animals (64).
Sheep and goats	We consider animals kept for meat production. Any acute loss of milk production due to disease is not considered. However, we do consider future lifetime milk production in the value of the animal.
Backyard indigenous chickens	This category describes indigenous breeds of chickens that are normally raised outdoors and allowed to range freely. Such chickens may be fed some feeds, or leftovers, but much of their nutrition comes from scavenging. Birds are not kept by the SSP for any particular period of time, unlike the broiler or layer systems which operate according to set production cycles (65, 66). While some of the products sold through this initiative may have been sold to broiler or layer production systems, the majority of product doses were sold to backyard farms.

#### 2.6.2. Data on production losses

To estimate the value of an animal to the SSP, baseline data on livestock production were obtained from the mean FAOSTAT production figures during the PL2 years of 2014–2017.^1^ To estimate the value of an animal death we took the mean carcass value, plus the discounted future milk and egg production, as the overall value of the animal. For validation, contemporaneous values for chicken sales were available from SSPs in project areas in India and Tanzania and they were in line with the FAOSTAT average (67, 68). They are estimated as presented in Table 4.

**Table 4:** List of economic parameters (values in USD) used in our model based on data fxrom FAOSTAT between 2014 and 2017.

	Cattle	Goat	Sheep	Small ruminants	Chickens
Sub Saharan Africa					
Total head (1000s)	*296,530*	*360,805*	*262,858*	*623,663*	1,204,975
Meat value per slaughtered animal	*$310.00*	*$24.62*	*$33.90*		*$2.46*
Milk (or egg) value per animal per year	*$179.14*	*$18.11*	*$11.69*		*$7.92*
Death - adult animal	*$350.22*	*$27.97*	*$36.36*	*$31.51*	*$3.62*
Death - young animal	*$175.11*	*$13.98*	*$18.18*	*$15.75*	*$1.81*
Death - overall (animal and milk/eggs)	**$315.19**	**$25.17**	**$32.73**	**$28.35**	**$3.25**
Loss of production per week	**$1.99**	**$0.47**	**$0.65**	**$0.56**	**$0.09**
South Asia					
Total head (1000s)	*273,038*	*300,246*	*152,742*	*452,988*	*3,229,441*
Value per animal	*$340.90*	*$41.40*	*$77.99*		*$3.96*
Milk (or egg) value per animal per year	*$578.23*	*$44.51*	*$13.83*		*$11.08*
Death - adult animal	*$542.66*	*$57.00*	*$81.72*	*$65.34*	*$5.30*
Death - young animal	*$271.33*	*$28.50*	*$40.86*	*$32.67*	*$1.59*
Death - overall (animal and milk / eggs)	**$488.39**	**$51.30**	**$73.55**	**$58.80**	**$4.55**
Loss of production per week	**$3.28**	**$0.80**	**$1.50**	**$1.15**	**$0.15**

Rows in italics are those that were used to derive the final estimates. The bold rows are the values used.

#### 2.6.3. Adjustment to present values

To adjust 2014–2017 economic values to present (2023) values we used the mean consumer prices index (CPI) inflation value from World Bank^2^ for the period 2014–2021 (2022 data were not available at the time of writing) for SA and Africa separately. We adjusted economic values and sales costs to present values according to the year and region of product sale.

### 2.7. Model implementation

We implemented the model to estimate the cost and benefits resulting from the sales of products under the GALVmed PL2 initiative.

Alongside the model presented in Figure 1, we also used a simple method for estimating the numbers of SSP customers reached by the initiative. The approach uses sales data and assumptions based on product pack sizes, typical buying patterns, and typical herd or flock sizes to estimate the total number of SSP customers ‘reached’ with a particular product. SSPs are disaggregated loosely into two broad segments in this approach:

1. SSPs with smaller sizes using mostly backyard systems (SSP-).
2. Larger, more extensive or commercialized SSPs (SSP+).

The customer number calculations are based on parameters that are defined by expert opinion for that target market. These include discussions with local veterinary experts, some internal GALVmed monitoring and evaluation data, and external expert input. The approach provides an approximation of the profile and magnitude of the customer base. This process is based on:

1. A pre-defined number of animals to describe the size of SSP- and SSP+ farms of different sizes (Table 5).
2. Each product sale is assigned to either the SSP- or SSP+ segment. For poultry products, we assume that 90% of doses were sold to SSP- and 10% to SSP+, for small ruminant products these proportions are 60 and 40% and for ECF vaccines 20 and 80% owing to pack size constraints with these products.
3. For each product, we estimate the number of doses required per farm per year.
4. We assume that there is overlap in terms of SSPs using multiple products within the same initiative. To avoid double counting, for ruminant products, we assume that 50% of farmers use a second product from the same initiative. Therefore, if the leading selling product to a species is 1,000, and there are 900 sales of the second best-selling product, then this is reduced to 450 to allow for multiple product purchases. For poultry products, where these products were typically sold concurrently, the percentage was 75%. These parameters vary depending on the range of products that are offered in a given initiative. Hence these parameters are illustrative.

**Table 5:** The average numbers of animals owned by SSP- and SSP+ used to estimate customer numbers.

	Animal numbers	
	SSP-	SSP+
Cattle	10	60
Small ruminants	20	100
Poultry (Africa)	25	1,000
Poultry (South Asia)	10	1,000

From these assumptions we broadly estimate a total number of customers reached.

To increase transparency, we implemented the model and the customer number estimations in Microsoft Excel (Supplementary material). Alongside this, we have presented sensitivity analyses in a version of the model that was implemented in R (69) with uncertainty analyses explored by a graphical user interface implemented in R-Shiny (70).^3^

### 2.8. Sensitivity analysis

We also conducted a range of sensitivity analyses that are intended to explore the heterogeneity in terms of how the diseases impact different regions or production systems, as well as variations in the specific nature of the disease owing to different strains. Specifically:

1. To explore the impact of potential ranges of the key parameters, we explore the NEB that results when each impact metric of mortality and meat production is adjusted between 50 and 200% of its baseline value. This is done for each disease.
2. We adjust the incidence of each disease between 50 and 200% of its baseline value and recalculate its gross economic benefit between those incidence rates.

## 3. Results

The gross economic benefit resulting from mortalities averted and production losses averted over the period of GALVmed’s PL2 initiative is $120.7 m (Table 6). With a total product sale cost of $15.5 m, this leaves a total NEB over the course of the initiative of $105.1 m (Table 6) across 3,664,114 customers, translating to an average of $28.54 per customer. This translates to $139.9 m in present values, and $37.97 per customer. ECF and ND vaccines have the greatest impact in terms of NEB. This is driven by very high sales of ND vaccines and high impact per animal treated for ECF–the latter due to the value of a bovine and the lifelong immunity arising from ECF vaccination.

**Table 6:** The results by product line.

Product	Region	Doses sold	2014–2017 values				Adjusted present net economic benefit	Estimated customers
			Sale cost (USD)	Total losses averted per dose (USD)	Total losses averted value (USD)	Net economic benefit (USD)		
PA	SA	8,191,251	265,397	0.36	2,032,094	1,766,697	2,418,815	46,104
PA	Africa	2,072,936	67,163	0.23	320,343	253,180	316,256	9,354
ND	SA	113,612,156	2,726,692	0.29	24,955,720	21,888,192	28,451,992	2,559,114
ND	Africa	55,501,674	1,332,040	0.20	8,522,666	7,024,121	9,041,050	467,976
FP	SA	3,084,167	74,020	0.25	681,982	598,709	796,285	34,735
FP	Africa	399,770	9,594	0.16	59,278	48,485	61,598	3,608
ECF	SA	–	–	–	–	–	–	
ECF	Africa	1,271,872	9,157,478	49.67	56,860,940	47,703,462	65,931,753	152,667
PPR	SA	2,754,833	396,696	5.18	12,834,338	12,437,642	15,837,338	46,832
PPR	Africa	2,013,837	289,993	2.51	4,551,905	4,261,912	5,228,288	136,941
SGP	SA	3,739,750	706,813	2.93	9,872,478	9,165,665	11,795,041	254,303
SGP	Africa	–	–	1.42	–	–	–	–
SGP & PPR	SA	–	–	8.18	–	–	–	
SGP & PPR	Africa	116,584	27,281	3.95	414,826	387,546	475,421	7,928
			**15,025,886**		**120,691,744**	**105,148,065**	**139,878,415**	**3,664,114**

PA, Poultry anthelminthics; ND, Newcastle disease; FP, Fowl pox; PPR, Peste des petits ruminants; SGP, Sheep and goat pox; ECF, East Coast fever; SA, South Asia.

Note that during the period the SGP & PPR vaccine was not available for sale in SA.

From the 3.66 m customers, the greatest numbers of customers reached was through ND vaccines in South Asia, owing to the very large numbers of doses (113.6 million) sold in this region. Similarly due to volumes of doses distributed ND vaccines have also covered large numbers of customers in Africa, as well as SGP vaccinated in SA where 254,303 customers are reached by 3.74 m dose sales.

**Figure 2:**
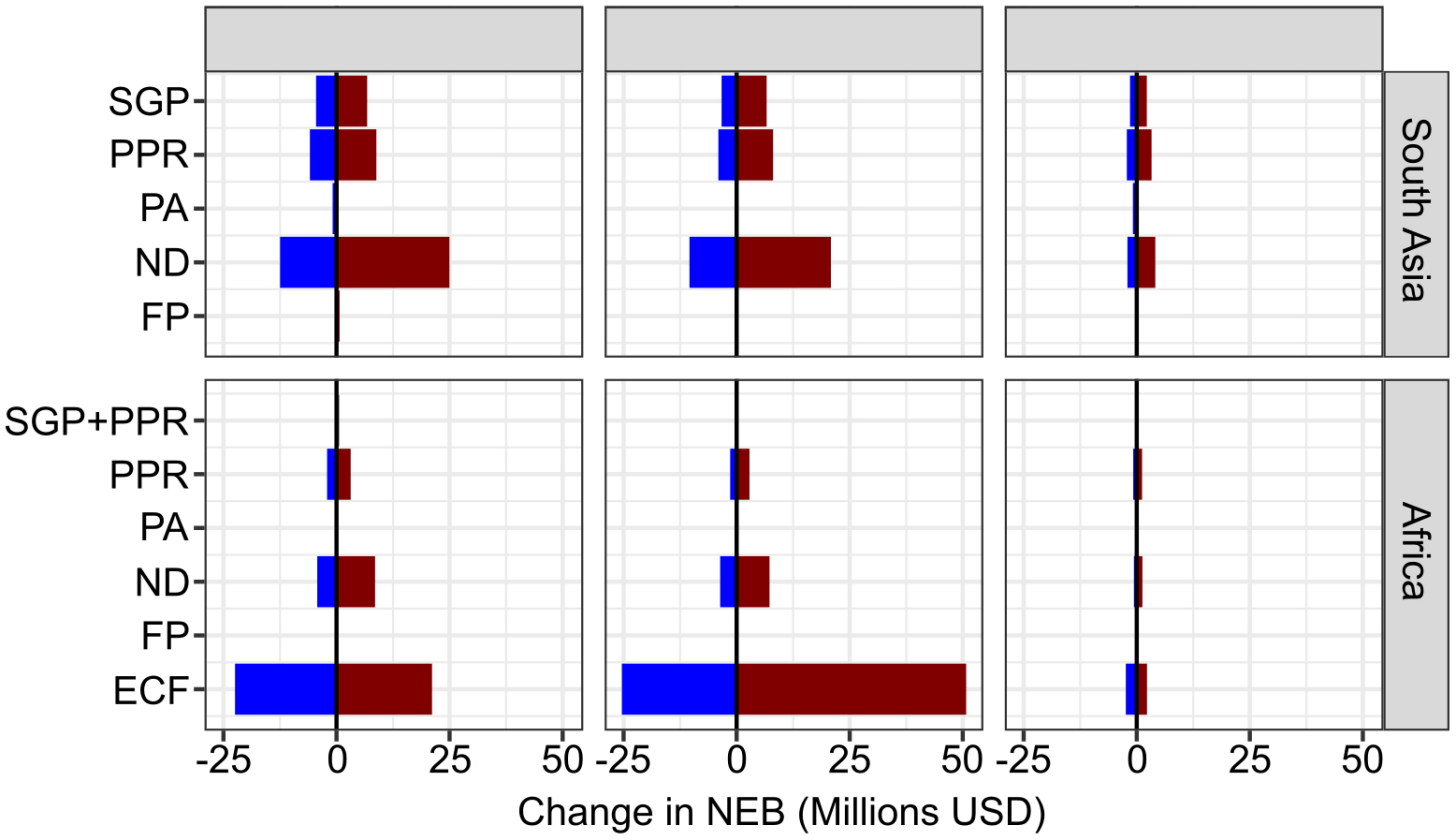
Tornado plot showing the change in NEB (x-axis) of changing the incidence values, mortality impact and production impacts between 50 and 200% of the baseline values. Blue indicating a negative change, red indicating a positive change.

Changing the disease between 50 and 200% of the baseline values shows that in Africa, ECF and ND are the most sensitive to local incidence, while in SA, ND is highly sensitive. ND is also sensitive in Africa, but in neither incidence geography are the other vaccines sensitive to the incidence (Figure 2). Varying the baseline mortality values, the ECF vaccines are also very sensitive, but ND vaccines remain highly sensitive to the parameter value (Figure 2). Sensitivity to productivity is less clearly impactful, but FP remains of lower importance in all areas, largely owing to the smaller number of doses that were sold (Figure 2).

## 4. Discussion

This model framework was developed for the specific purpose of evaluation and decision-making on product development and product impact in GALVmed’s work with SSPs. In order to serve as a strategic decision making tool within GALVmed, the approximate estimates of impact generated provides a sufficiently clear picture by which the necessary strategic comparisons and decisions can be made. As this framework is designed to work at the macro level–at the level of countries or regions, encompassing all the relevant SSP production types and evolving product portfolios - it is necessary to keep the framework simple and flexible to incorporate new products as necessary. Therefore, the model is not a static platform; rather it is one that can be evolved and improved as new products are added and new parameter estimates become available. The framework could be used in a similar way to estimate the impact of other programs designed to improve the access to and utilization of animal health products in SSP settings. Furthermore, it can be tailored and used in a prospective way to estimate the potential impact of a planned intervention or to identify target areas for interventions.

The long term aim of the programs that GALVmed undertakes and looks to evaluate, is to see a transformational improvement in the economic progression and wellbeing of SSPs in Africa and SA by securing sufficient access for SSPs to key animal health products through market-led initiatives. Market development initiatives yield accurate sales data and therefore this is the starting point for the model. Here we have applied this model framework to demonstrate that the GALVmed market development initiatives between 2014 and 2017 generated an estimated $105.1 m (in 2014–2017 values; $139.9 m adjusted) in net economic benefits to the estimated 3.7 m SSPs that were reached through the initiatives. This translates to an average of $28.54 per customer reached ($37.97 adjusted), reflecting the predominance of backyard poultry farms among the sales where we are assuming relatively small farms and therefore lower scope for impact per SSP. The results are highly sensitive to the local incidence rates of the disease as well as to the mortality rate, with ECF varying by up to $50 m USD at the upper limits, underlining the importance of local disease situation and aspects that can influence mortality such as breed and pathogen strain (71). While this was used here as a tool to measure the impact following the initiative, it can be used in future as a tool both to forecast the potential impact and then measure the progress toward that impact. While here ND and ECF vaccine sales have had the greatest impact in terms of NEB, this is in part due to the composition of the initiative and the emphasis placed on these products. In planning future initiatives this could be considered to strike an optimal balance between the potential to deliver sales and produce impact.

Other modeling approaches have developed metrics for comparison between, and prioritization of diseases as well as for analysis of gaps in disease knowledge (72–74) and modeled global losses from diseases (15). A slightly different approach is adopted by the Global Burden of Animal Diseases (GBAD) loss envelope (30) which looks at differences between maximum possible production and real production, then divides the gap up by different causes (diseases etc.). While the model presented here is a similar loss-envelope approach, our starting point is sales of particular products, and we consider each disease in isolation rather than as a holistic production system.

Individual studies have modeled the benefit–cost ratios of interventions against bovine trypanosomiasis and against tsetse fly vectors (75, 76). These provide detailed estimations for a specific disease, but the estimates cannot easily be transferred to other diseases. Additionally, there are estimations of economic losses incurred due to specific diseases in the same geographies as our analysis. Annual losses from PPR in South Asia were forecast at $2.9 billion per year for the period 2012–2017 (53). The global net benefits of PPR eradication were estimated to be $74.2billion with a benefit cost ratio of 34:1 (52). Here, we estimated $8 million losses averted from 2.75million vaccine dose sales in South Asia. While these estimates for individual diseases illustrate the impacts of diseases, and support our conclusions around the benefits of interventions, they do not give a benchmark for the full selection of interventions modeled here. For the model framework presented here, there is no known suitable comparison model.

The model is intended as a practical tool and, as a consequence of the simplicity of the framework, there are broad assumptions that underpin the model mainly due to the gaps in obtaining reliable data and parameter estimates. The model considers only the direct costs and benefit to the SSP. Ultimately, the willingness to pay for products represents a farm management or investment decision as to the return on investment in the animal health product versus the counterfactual productivity burden if no product is given (17, 77). The simplicity of the framework owes largely to fact that it is applied at a high level and over a large scale to compare market and product development initiatives. The model incorporates a number of limitations owing to its simplicity:

1. An average NEB estimate for a vaccine is very simplistic and does not consider that, in reality, the financial gain or loss will be binary. For SSPs, the vaccine would either avert an outbreak of disease in which case there would likely be substantial positive benefit or would not avert an outbreak (because the animals were not exposed) in which case the SSP would make a small loss of the cost of purchasing the vaccine.
2. The model is essentially a temporal snapshot and does not consider any of the longer term dynamics of farming changes subsequent to preventing disease.
3. The within-animal separation is relatively simple. There is little differentiation between juvenile and adult animals, or the specific uses of the animals.
4. The model does not differentiate between breeds, whereas there are important differences existing between the productivity of different breeds and their susceptibility to disease (52, 71, 78).
5. Any potential subsequent costs are not considered. For example, in the case of a disease such as ECF, we assume that for ECF the unvaccinated animal will die with a certain probability and do not consider the possibility of the infected animal being treated. However, some of these animals will be treated which will give the animal a certain probability of survival and thus offset the loss, but at a cost (59, 79). This averted cost is not measured here.
6. Related to 5, we do not consider ongoing interventions which may be reduced such as reduced acaricide use in ECF endemic areas (71).
7. We do not consider the wider health of animals with greater levels of disease incidence and disease severity when animals are more stressed due to co-infection, malnutrition, or dehydration (or commonly all).
8. We treat product efficacy for vaccines as a continuum whereas for some diseases, such as ND, it may be closer to binary with an entire flock or population not protected against a circulating strain.
9. We do not account for variations in the life spans of animals and variations in the amount of time that animals are held for.
10. The model assumes that all products are given to different animals and no adjustment is made for a single animal receiving multiple products.

Additionally, we have modeled the impacts on certain key product classes where the impacts are relatively easy to model because their impact is relatively easy to estimate. However there are key products such as acaricides and antibiotics that could also be modeled and impact estimated. Acaricides present a challenge because they are treating a direct problem (the arthropods) and preventing the indirect problem (tick borne diseases) and secondary infections resulting from the ticks and efforts to deal with them (for example infected lesions from scratching) (80, 81). Disentangling these problems and estimating impacts presents unique challenges. Antibiotics present a different challenge in that most compounds are non-specific and can treat a range of conditions or are often used on a syndromic basis rather than following a specific diagnosis.

In implementing this model for the PL2 initiative, we assume that products were primarily sold to small SSPs (SSP-). This is particularly pertinent to poultry products, where the production dynamics for commercial broiler and layer systems are quite different to backyard indigenous systems. In the commercial systems, the throughput is much greater, and products are used at a higher rate than backyard systems. The production systems could be refined in future to include new systems or to add further detail. In particular, the model could be refined to include the differences in production between SSP- and SSP+, particularly to include production differences with improved animal breeds.

The sensitivity analyses have shown that the NEB is sensitive to changes in disease incidence and mortality impact. Thus, as the parameters change in different geographies, the local impacts and estimated benefits would vary substantially. If the model were applied to a specific geography with known disease incidence rates, or economic values, then the model could be updated accordingly. We did not model sensitivity to product cost because these were actual values from these initiatives rather than variable parameters. Were the model to be used to estimate the impact of other initiatives or to estimate potential returns from a planned initiative, then these input cost parameters could be adjusted or explored for sensitivity.

The sensitivity analyses highlights that this is a program-level tool for evaluating animal health interventions to inform national and international level stakeholders. The sensitivity analysis shows how this can vary locally depending on the local disease and livestock situation. Advising SSPs on the benefits of interventions at the local level should be based on local veterinary expertise, or specific studies carried out that can take into account the local epidemiological, ecological, and agricultural picture and should not be based on a high level model such as this.

The model can be further augmented and updated as new parameters are published from GALVmed’s field studies and from bespoke validation exercises to validate key assumptions. In addition, a core objective of SEBI-L is to improve the consumption and utilization of existing data through systematic reviews of evidence supported by novel informatics approaches (that cut down the time taken for such reviews). These reviews can be employed to improve the model parameter estimations.

Here we have presented a flexible modeling framework to evaluate the impact of implementing market development initiatives for expanding the uptake of animal health products. The framework aims to give a broad overview estimate and be entirely transparent and adaptable to new initiatives that are implemented by GALVmed or other NGOs. As an evolving approach, future improvements both to the model’s assumptions as well as to the model structure itself will be made based on some of the limitations noted in this paper. As GALVmed’s initiatives change and evolve, so too does the model need to evolve to remain relevant.

Following the PL2 initiative, the model is being extended to consider the impacts of further initiatives, including interventions targeted at diseases that acutely impact milk or egg production, and reproduction. Further changes to the model will be undertaken to enable as accurate and encompassing an estimation of impact as possible as GALVmed’s initiatives change and develop over time.

## Data availability statement

The original contributions presented in the study are included in the article/Supplementary material, further inquiries can be directed to the corresponding author.

## Author contributions

PB, GS, AP, LA-R, and KT conceived the model. PB and GS designed the model and compiled data. CS conducted sensitivity analysis. PB, CS, GS, and AP drafted the paper. All authors contributed to the article and approved the submitted version.

## Funding

This publication is based on research funded in part by the Bill & Melinda Gates Foundation (Investment ID OPP1176784) and with UK aid from the UK Government (Project 300504) through GALVmed. The findings and conclusions contained within are those of the authors and do not necessarily reflect positions or policies of the Bill & Melinda Gates Foundation or the UK Government, but a draft of the manuscript was reviewed by the Bill & Melinda Gates Foundation.

## Conflict of interest

The authors declare that the research was conducted in the absence of any commercial or financial relationships that could be construed as a potential conflict of interest.

## Publisher’s note

All claims expressed in this article are solely those of the authors and do not necessarily represent those of their affiliated organizations, or those of the publisher, the editors and the reviewers. Any product that may be evaluated in this article, or claim that may be made by its manufacturer, is not guaranteed or endorsed by the publisher.

## Footnotes

^1^ https://www.fao.org/faostat/en/

^2^ https://data.worldbank.org/indicator/FP.CPI.TOTL.ZG

^3^ https://sebi-livestock.shinyapps.io/Impactmodel/

## Supplementary material

SUPPLEMENTARY INFORMATION S1 Exemplar model showing the working underpinning the model that produced the results presented here.
